# The Effectiveness of the Be Prepared mHealth App on Recovery of Physical Functioning After Major Elective Surgery: Multicenter Randomized Controlled Trial

**DOI:** 10.2196/58703

**Published:** 2025-05-30

**Authors:** Miriam van der Velde, Marike van der Leeden, Edwin Geleijn, Cindy Veenhof, Karin Valkenet, Be Prepared Group, Ruben PA van Eijk

**Affiliations:** 1 Research Group Innovation of Human Movement Care, Center for Healthy and Sustainable Living, HU University of Applied Sciences, Heidelberglaan 7, Utrecht, 3264 LJ, The Netherlands, 31 0642214251; 2 Department of Rehabilitation, Physical Therapy Science and Sports, University Medical Center Utrecht, Utrecht University, Utrecht, The Netherlands; 3 Department of Rehabilitation Medicine, Amsterdam University Medical Center, Vrije Universiteit Amsterdam, Amsterdam, The Netherlands; 4 Amsterdam Public Health Research Institute, Amsterdam University Medical Center, Vrije Universiteit Amsterdam, The Netherlands

**Keywords:** perioperative care, mHealth, prehabilitation, surgery, digital health

## Abstract

**Background:**

Patients undergoing major surgery are at risk of complications and delayed recovery. Prehabilitation has shown promise in improving postoperative outcomes. Offering prehabilitation by means of mHealth can help overcome barriers to participating in prehabilitation and empower patients prior to major surgery. We developed the Be Prepared mHealth app, which has shown potential in an earlier pilot study.

**Objective:**

This study aims to evaluate the effectiveness of the Be Prepared app on postoperative recovery of physical functioning (PF) in patients undergoing major elective surgery.

**Methods:**

This study was a multicenter randomized controlled trial with 2 arms. Adults scheduled for major elective surgery were randomly assigned to the control (usual care) or intervention group (Be Prepared app in addition to usual care). The Be Prepared app is a smartphone app with pre- and postoperative information and instructions on changing risk behavior for patients undergoing major elective surgery. The primary outcome was recovery of postoperative PF up to 12 weeks after hospital discharge measured with the Computer Adaptive Test Patient-Reported Outcomes Measurement Information System-PF. Secondary outcomes included social participation, self-reported recovery, health-related quality of life, postoperative outcomes, and patient satisfaction. Measurements were performed at 5 time points: before random assignment and 1, 3, 6, and 12 weeks after hospital discharge.

**Results:**

A total of 369 patients were analyzed, 181 in the control group and 188 in the intervention group. The result of the linear mixed effects model showed a mean slope difference in recovery of PF over 12 weeks of 2.97 (95% CI 0.90-5.02) in favor of the intervention group. However, this effect was not clinically relevant and was negated by the significantly lower PF score 1 week after hospital discharge in the intervention group (mean difference –1.72, 95% CI –3.38 to –0.07). Most secondary outcome measures did not show significantly greater improvements in the intervention group compared to the control group. Patient satisfaction with overall perioperative care was significantly higher in the intervention group compared to the control group and satisfaction with the Be Prepared app was high.

**Conclusions:**

The use of the Be Prepared app as a stand-alone intervention does not seem beneficial for improving postoperative recovery in patients undergoing major surgery. However, satisfaction with perioperative care was higher in patients using the app. Given the advantages of digital technology in health care, it can be considered a basis for prehabilitation care pathways, complemented by guidance from health care professionals as needed.

## Introduction

Patients undergoing major surgery are at risk of adverse postoperative health outcomes such as complications and delayed or poor recovery [1,2]. There is growing evidence showing that prehabilitation can improve postoperative recovery and reduce the incidence of postoperative complications [1-6]. Prehabilitation aims to enhance the patients’ general health and well-being prior to major surgery by modifying behavioral and lifestyle risk factors [3]. With prehabilitation, patients can better withstand the forthcoming stressor of surgery and thus minimize the risk of postoperative complications and prolonged hospital stay and enhance recovery after surgery [2-4,6]. For patients, recovery of physical functioning (PF) and well-being are the most highly valued goals following surgery [7].

Prehabilitation is preferably multimodal and involves the optimization of the physical, nutritional, and mental status of patients undergoing major elective surgery [2,4,6]. Prehabilitation interventions can vary widely, including in terms of modalities, context (home-, community-, or hospital-based), target population, and degree of supervision. Qualitative evidence shows that many patients undergoing major surgery prefer home-based prehabilitation [4,8,9]. Home-based prehabilitation helps overcome various barriers to participation in prehabilitation: it resolves transportation and parking issues, it makes it easier for patients to combine prehabilitation with their everyday life, and patients with physical or psychological symptoms might be more comfortable at home [8]. Using a mobile health (mHealth) app as a home-based strategy for prehabilitation could be an effective approach and is in line with current developments in health care in which digital technologies play a crucial role in providing sustainable, efficient, and patient-centered health care [10,11]. In addition, mHealth can positively influence self-efficacy and empower patients prior to major surgery [12-14].

We developed the Be Prepared mHealth app for patients undergoing major surgery. The Be Prepared app is a smartphone app with pre- and postoperative information and instructions to prepare patients for major surgery. The app is multimodal and focuses on changing risk behavior (ie, smoking and alcohol cessation, increasing physical activity and muscle strengthening activities, and protein-rich food consumption). The first version of the Be Prepared app has proven potential in a pilot study in terms of usability, patient satisfaction, and modifying risk behavior prior to surgery [15]. Several points of improvement were identified in this pilot study and have been addressed as part of the further development of the app prior to this study. These included expanding the preoperative content, extending the content postoperatively, and further personalization of the Be Prepared app [15].

The primary aim of this multicenter randomized controlled trial (RCT) was to evaluate the effectiveness of the Be Prepared mHealth app on the recovery of postoperative PF, when added to usual care, in a population of patients undergoing major elective surgery. The secondary aim was to evaluate the effectiveness of the Be Prepared mHealth app on social participation, self-reported recovery, health-related quality of life, postoperative outcomes, and patient satisfaction.

## Methods

### Design and Participants

This study was conducted as a single-blind parallel multicenter RCT. Patients were eligible for participation if they were scheduled for elective surgery with an expected postoperative hospital stay of at least 2 nights. Patients had to be aged 18 years or older and have one or more risk behaviors (ie, currently smoking, ≥7 alcohol consumptions per week, moderate-intensity physical activity <30 min every day, muscle-strengthening activities on <2 days a week, and unintentional weight loss of >3 kg in the last month). Patients were excluded if they had insufficient comprehension of the Dutch language, had no access to a mobile device, had to undergo brain surgery, had their surgery scheduled within 7 days, were participating in conflicting studies, or in an intensive preoperative care pathway (eg, an exercise program).

### Recruitment and Blinding

Four centers participated in the trial: 3 university hospitals and 1 general hospital in the Netherlands. Potentially eligible patients were identified at the preoperative assessment at the participating hospitals and received an invitation letter to participate in the study. After providing informed consent and completing the baseline questionnaire, patients were randomly assigned in a 1:1 ratio to the control group (usual care) or the intervention group (usual care plus the Be Prepared mHealth app) using a web-based randomization system. All patients received an automated email of their group allocation. Patients were aware of their group allocation, but researchers analyzing the data and health care professionals at the hospital were kept blinded to the allocation. Patients in the intervention group filled in the Physical Activity Readiness Questionnaire (PAR-Q) before starting the intervention to determine whether they could safely perform physical activity [16,17]. Patients who were scheduled to receive instructions on increasing physical activity and muscle strengthening through the app were contacted by a member of the research team when answering yes to one or more questions in the PAR-Q. These patients were advised, when necessary, to contact their physician before commencing the intervention.

### Intervention

All patients received usual care. Usual care varied widely, depending on hospital guidelines and the respective care pathway. In most cases, preoperative care consisted of a preoperative consultation with the anesthesiologist (digital, by telephone, or face-to-face) and information about general preoperative preparation through leaflets. A single care pathway, for example, the esophageal cancer care pathway, includes a preoperative consultation with a dietician or a physiotherapist. Counseling on smoking or alcohol cessation is not a standard part of any care pathway.

Patients in the intervention group had access to the Be Prepared mHealth app (Patient Journey platform by Interactive Studios BV), in addition to usual care. After randomization, patients received an automated email with a link to download the app. The Be Prepared app is a smartphone app with pre- and postoperative information and instructions on changing risk behavior for patients undergoing major surgery. The Be Prepared app was deployed as a stand-alone intervention, and no health care professionals were involved during the use of the app. The content presented in the app was tailored to the patients’ risk profile, meaning patients only received content on the risk behaviors present. At the start of the intervention and again after hospital discharge, patients answered screening questions in the app, regarding risk behavior, to determine their risk profile. The content of the app differed according to the risk profile, and focused on smoking and alcohol cessation, increasing physical activity and muscle-strengthening activities according to the recommendations of the Dutch Health Council, and protein-rich food consumption. Furthermore, the mHealth app included content on general preparation (eg, preoperative fasting and medication intake prior to surgery), stress, and practical issues (eg, transportation and postoperative care).

Patients were advised to use the app in the run-up to their surgery, during their hospital stay, and in the first weeks after hospital discharge. The day-to-day information and instructions were presented on a dynamic timeline based on the patient’s operation date. The timeline provided written information, information videos, patient-experience videos, tips on healthy behavior and changing risk behavior, quiz questions, and exercise videos. Several behavior change techniques were used in this app, such as goal setting and feedback on behavior [18]. Push notifications informed the patient about available new content. The amount of content depended on the patients’ risk profile.

**Figure 1.**
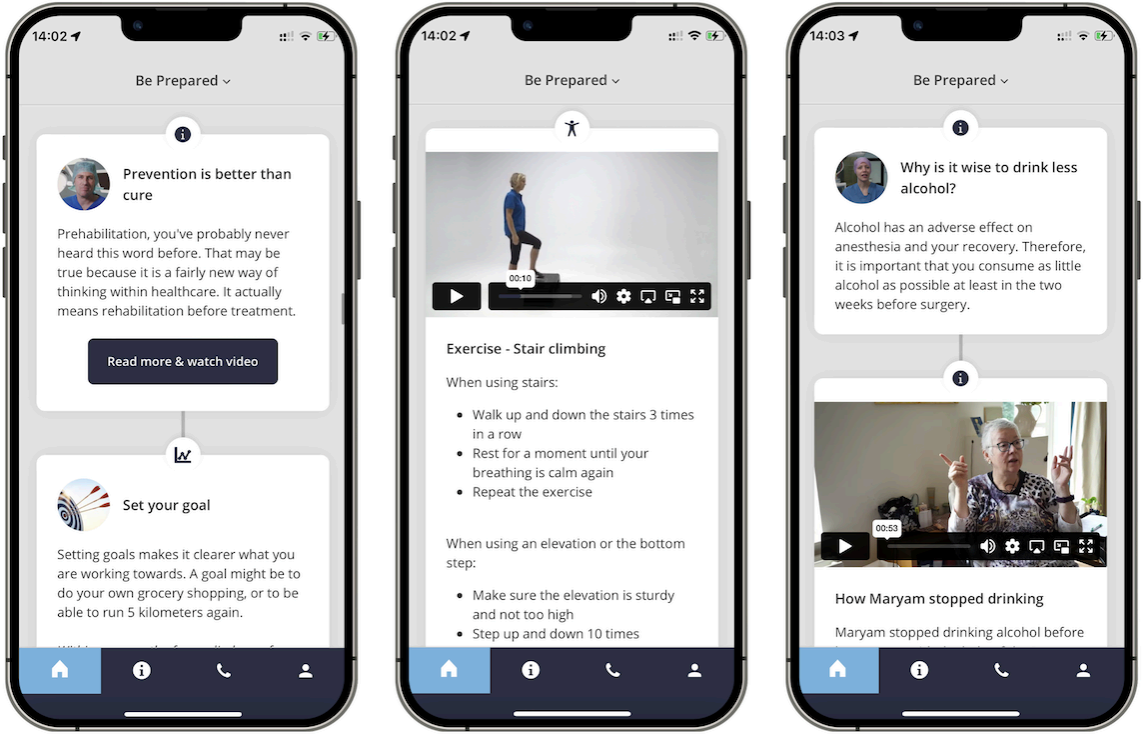
Screenshots of the Be Prepared app (translated from Dutch).

Furthermore, participants were asked whether they succeeded in following the instructions and received automated feedback based on their responses. When patients repeatedly were unsuccessful in following the instructions, they were advised to contact a health care professional of their choice for personal counseling; there were no health care professionals affiliated with the study. In addition, the patient’s feedback was used to tailor exercises to the patient’s level. Based on the patient’s feedback, the level of exercises was maintained, scaled up, or scaled down. Data collected by the app and app usage are stored anonymously in a database of Interactive Studios (ISO 27001 and NEN7510 certified). There was no integration with the hospital’s electronic health record. Screenshots of the app can be found in Figure 1. An overview of the intervention content and features can be found in Multimedia Appendix 1.

### Compliance

Through user authentication, the use of the mHealth intervention was registered by date and time: for example, opening the app, answering in-app questions, and opening information. Self-reported app use was registered by asking patients in the web-based questionnaire if they had used the app regularly, occasionally, or not at all.

### Outcome Measures

Patients completed online questionnaires at 5 time points: before random assignment (T0), 1 week (T1), 3 weeks (T2), 6 weeks (T3), and 12 weeks (T4) after hospital discharge. The timing of the baseline measurements (T0) relative to the surgery date varied. At baseline, demographic and clinical characteristics were collected through questionnaires and electronic health records (ie, age, sex, BMI, the American Society of Anesthesiologists (ASA) physical status classification, surgical specialty, waiting time for surgery, risk behaviors, level of education, and Charlson Comorbidity Index).

The primary outcome was the recovery of PF (from 1 to 12 weeks after hospital discharge) assessed by the Computer Adaptive Test (CAT) Patient-Reported Outcomes Measurement Information System-Physical Function (PROMIS-PF). The CAT PROMIS-PF is a computer-administered measure with questions that are selected by a computer algorithm, based on a patient’s response and their estimated health state, from the PROMIS-PF item bank (version 1.2). The item bank contains 121 items, covering a wide range of (everyday) activities from self-care to more complex activities. Standard PROMIS CAT stopping rules were used [19]. The minimal important difference (MID) was estimated using a distribution-based model (0.5 SD at baseline) [20]. The estimated MID for this sample was 4.18.

The secondary outcomes included PF, social participation, self-reported recovery, and health-related quality of life at the different time points after surgery, the course of social participation, self-reported recovery, and health-related quality of life, in-hospital physical and mental symptoms, self-reported risk behavior change, in-hospital mobilization, postoperative outcomes, and patient satisfaction (Table 1). Scores on CAT PROMIS-PF, CAT PROMIS Ability to Perform Social Roles and Activities (APS), and European Quality of Life 5 Dimensions 3 Level Version were calculated according to published scoring algorithms [19,21-23].

**Table 1.** Primary and secondary outcome measures.

Outcome	Instrument or method	Item information	Scoring	Interpretation	Time points^a^
Primary outcome measure					
PF^b^	CAT^c^ PROMIS-PF^d^ (v1.2) [19,21]	4-12 questions from the item bank containing 121 items 5-point response scale from 1 to 5	T-score metric (mean 50, SD 10)	Higher is better outcome	T0, T1, T2, T3, T4
Secondary outcome measures					
Social participation	CAT PROMIS-APS^e^ (version 2.0) [21]	4-12 questions from the item bank containing 35 items 5-point response scale from 1 to 5	T-score metric (mean 50, SD 10)	Higher is better outcome	T0, T1, T2, T3, T4
Self-reported recovery	Self-reported recovery (study specific)	11-point response scale from 0=not recovered to 10=fully recovered	0‐10	Higher is better outcome	T1, T2, T3, T4
Health-related quality of life	EQ-5D-3L^f^ [22,23]	5 items Response scale from 1=no problems to 3=extreme problems	Health state index score 0‐1	Higher is better outcome	T0, T1, T2, T3, T4
In-hospital physical and mental symptoms	In-hospital physical and mental symptoms (study specific)	3 items on physical symptoms 5-point response scale from never to extremely often	0‐12	Lower is better outcome	T1
		4 items on mental symptoms 5-point response scale from never to extreme often	0‐16		
In-hospital mobilization	In-hospital mobilization (study specific)	3 items: sitting in chair, hospital room ambulation, and hallway ambulation 6-point response scale from 0=day of surgery to 5=day 5 or later.	0‐15	Lower is better outcome	T1
Self-reported risk behavior change	Risk behavior change: stopped smoking, stopped drinking alcohol, increased physical activity, increased muscle strengthening activities, and increased protein rich food consumption. (2-point scale; yes-no; study specific)	5 items Binary response scale (yes or no)			T1
Postoperative outcomes	Total and severe postoperative complication rates (EHR^g^)	Severity of complications are graded according to Clavien-Dindo classification Grade I (least severe) to grade V (death) [24]	I-V	Clavien-Dindo grade III or higher is classified as a severe complication	Within 30-days after surgery
	Length of hospital stay (EHR)	Days between admission and discharge from hospital			
	Hospital readmission rates (EHR)				Within 30-days after surgery
Patient satisfaction	Satisfaction with perioperative care	NRS^h^ from not satisfied at all to very satisfied	0‐10	Higher is better outcome	T1
	Satisfaction with the Be Prepared app (intervention group)	NRS from not satisfied at all to very satisfied	0‐10	Higher is better outcome	T1
	Net Promotor Score [25] (intervention group)	Rating between 0 and 10 The score is calculated by subtracting % of detractors (0‐6) from % of promotors (9-10)	−100 to 100	Positive total NPS is good	T1

a T0: before random assignment, T1: 1 week, T2: 3 weeks, T3: 6 weeks, and T4: 12 weeks after hospital discharge.

b PF: physical functioning.

c CAT: Computer Adaptive Test.

d PROMIS-PF: Patient-Reported Outcomes Measurement Information System-Physical Functioning.

e PROMIS-APS: Patient-Reported Outcomes Measurement Information System-Ability to Perform Social Roles and Activities.

f EQ-5D-3L: European Quality of Life 5 Dimensions 3 Level Version.

g EHR: electronic health record.

h NRS: Numeric Rating Scale.

### Sample Size

A sample size calculation was performed on the primary outcome: the postoperative recovery of PF. To achieve a power of 0.85, with a 2-sided significance level of .05, 402 patients would be needed (201 per arm) to detect a mean standardized effect of 0.30 (ie, small to moderate effect) between the intervention and usual care according to an independent Student *t* test (2-tailed). As patients are assessed at multiple time points and analyzed accordingly, thus increasing the amount of information, the estimated sample size is conservative. In addition, we assumed a dropout rate of 0.15 and aimed to include 480 patients.

### Statistical Analysis

Characteristics of the study population are summarized per treatment group using descriptive statistics. Primary and secondary outcomes were analyzed according to the modified intention-to-treat principle, meaning that we only included participants who completed at least 1 follow-up measurement [26,27].

The differences between groups on the postoperative course of the primary and secondary longitudinal outcomes were analyzed using linear mixed effects models. The fixed part of the models included a term for the baseline score of the respective outcome (except for the model for self-reported recovery), time, group allocation, and the interaction between group allocation and time. The random part of the model contained a random intercept and slope for time per individual. Treatment effectiveness was determined by the statistical significance of the time by group interaction, using a likelihood ratio test with 1 degree of freedom, indicating whether the rate of change over time was different in the intervention or control group. The difference in rate of change over time has been expressed as a difference in change over 12 weeks to ease interpretation. No imputation techniques were used, as missing data were addressed by the chosen modeling strategies.

Three sensitivity analyses were conducted for the primary outcome. To explore the impact of missing data, the primary outcome, in participants without any follow-up measurement, was once imputed with the lowest score and once with the highest score measured at that time point. To assess possible center effects, we performed a sensitivity analysis, including a 3-way interaction between group allocation, time, and center, which was assessed using a likelihood ratio test [28]. In addition, we performed a per-protocol analysis for the primary outcome in which only data from participants who activated the app, answered the in-app screening questions, and used the app at least once after the first login, were included.

A mixed-effects model for repeated measures was used to analyze the between-group differences in the longitudinal outcomes at each postoperative time point using similar random and fixed effects.

We estimated odds ratios (ORs) and their CIs between the intervention and control group of the binary outcomes (overall complications, severe complications, and hospital readmissions) using logistic regression. A Cox proportional hazards model was used to estimate hazard ratios and their CIs for length of hospital stay. A Mann-Whitney *U* test was performed to compare the in-hospital physical and mental symptoms sum scores, in-hospital mobilization sum scores, and patient satisfaction with perioperative care. A chi-square test was used to compare self-reported risk behavior change between groups.

Subgroup analyses were performed, on the primary outcome, to explore if the effects of treatment might differ across specific groups of patients. Subgroup analyses were specified a priori in the study protocol and are exploratory and hypothesis-generating. We compared the following subgroups: intervention duration (<14 days, 14‐28 days, or >28 days), surgery type (oncological or nononcological), and number of risk behaviors (≤2 risk behaviors or ≥3 risk behaviors).

Analyses were performed using IBM SPSS for Windows version 28.0 and RStudio (version 2022.07.2; R Foundation for Statistical Computing). A significance level of α=.05 was applied for all tests.

### Ethical Considerations

Written informed consent was obtained from all participating patients. This study has been approved by the Medical Ethical Committee of Amsterdam University Medical Center, location VUmc, (registration NL61503.029.18), and has been registered at the Overview of Medical Research in the Netherlands (NL-OMON53078). Overview of Medical Research in the Netherlands is an official data provider to the International Clinical Trial Registry Platform of the World Health Organization. All data collected in the trial were deidentified and stored in a secured cloud storage environment. There was no compensation for the participants. The CONSORT (Consolidated Standards of Reporting Trials) guidelines were followed.

## Results

### Participant Flow

**Figure 2.**
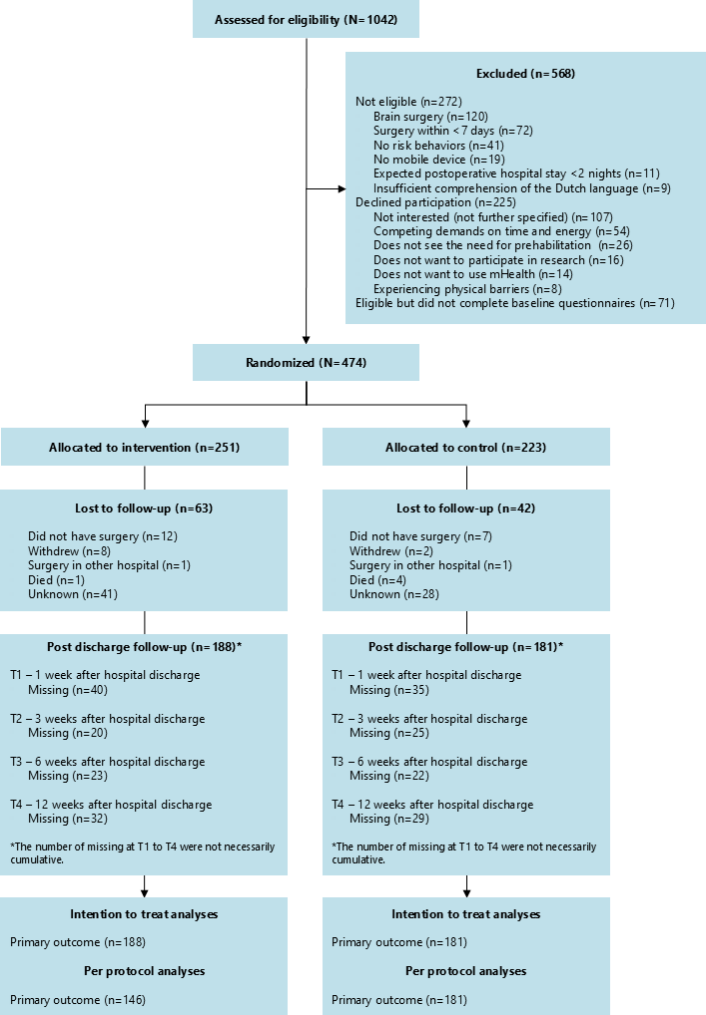
Flow of participants through the trial.

Between June 2020 and June 2022, 1042 patients were assessed for eligibility. In total, 225 patients declined participation and 272 patients did not meet the inclusion criteria. The reasons for nonparticipation are mentioned in Figure 2. Furthermore, 71 patients were eligible but did not complete the baseline questionnaire and were therefore not randomized. Finally, 474 patients were randomly assigned to the intervention (n=251) or control (n=223) group of whom 369 (78%) completed at least 1 follow-up questionnaire and were therefore included in the final analyses (188 patients in the intervention group and 181 in the control group). When comparing characteristics of participants included in the analysis (N=369) to those who only completed baseline questionnaires (n=105), the participants not included in the final analyses scored lower on the baseline scores of the CAT PROMIS-PF (mean difference 3.21, 95% CI 1.36-5.03) and CAT PROMIS-APS (mean difference=3.57, 95% CI 1.26-5.87) and more frequently had an ASA classification of 4 (7% vs 1%).

### Patient Characteristics

Participants had a mean age of 62 (SD 11.9) years, 60.4 % were male (223/369), and 41.5% (153/369) had a high level of education. Most participants underwent gastrointestinal surgery (124/369, 33.6%) or cardiothoracic surgery (103/369, 27.9%). The median waiting time for surgery was 27 (IQR 14‐51) days. Patient characteristics per group are shown in Table 2. The number of included patients per participating center varied between 7 and 246. No intervention-related adverse events were reported during the trial.

**Table 2.** Baseline characteristics of participants.

	Modified intention-to-treat	
Characteristics	Intervention (n=188)	Control (n=181)
Age (years), mean (SD)	63.3 (11.3)	60.1 (12.5)
Sex (male), n (%)	111 (59)	112 (61.9)
BMI (kg/m^2^), median (IQR)	25.4 (23.2-29.1)	25.5 (23.4-28.7)
ASA^a^ classification, n (%)		
I	14 (7.4)	14 (7.7)
II	87 (46.3)	93 (51.4)
III	76 (40.4)	63 (34.8)
IV	3 (1.6)	2 (1.1)
Unknown	8 (4.3)	9 (5)
Surgical specialty, n (%)		
Gastrointestinal	58 (31)	66 (37)
Cardiothoracic	55 (29.3)	48 (26.5)
Urology and gynecology	29 (15.4)	20 (11)
Orthopedic and spine	21 (11.2)	13 (7.2)
Head and neck	10 (5.3)	20 (11.0)
Vascular	10 (5.3)	9 (5)
Plastic	4 (2.1)	4 (2.2)
Neurosurgical	1 (0.5)	1 (0.6)
Surgical oncology, n (%)	99 (52.7)	101 (55.8)
Waiting time for surgery^b^ (days), median (IQR)	26 (14-53)	27 (14-50)
Risk behaviors^c^, n (%)		
Smoking	16 (8.5)	16 (8.8)
Alcohol consumption	20 (10.6)	12 (6.6)
Physical inactivity	145 (77.1)	144 (79.6)
No muscle strengthening activities	173 (92)	173 (95.6)
Unintentional weight loss	25 (13.3)	24 (13.3)
Number of risk behaviors, n (%)		
1	29 (15.4)	25 (13.8)
2	133 (70.7)	130 (71.8)
3	21 (11.2)	20 (11)
4	4 (2.1)	6 (3.3)
5	1 (0.5)	—^d^
Level of education^e^, n (%)		
Low	46 (24.5)	50 (27.6)
Intermediate	68 (36.2)	45 (24.9)
High	70 (37.2)	83 (45.9)
Unknown	4 (2.1)	3 (1.7)
CCI^f^, n (%)		
Mild (1-2)	96 (51.1)	93 (51.4)
Moderate (3-4)	35 (18.6)	24 (13.3)
Severe (≥5)	15 (8)	18 (9.9)
CAT^g^ PROMIS-PF^h^, mean (SD)	44.94 (8.36)	45.68 (8.35)
CAT PROMIS-APS^i^, mean (SD)	50.50 (11.08)	49.91 (11.02)
EQ-5D-3L^j^, mean (SD)	0.79 (.20)	0.83 (.18)

a ASA: American Society of Anesthesiologists.

b Days between baseline measurement and surgery.

c Multiple response options.

d Not applicable.

e Low: preschool, primary school, lower vocational education; intermediate: secondary education, intermediate vocational education; high: higher vocational education, university, postgraduate.

f CCI: Charlson Comorbidity Index.

g CAT: Computer Adaptive Test.

h PROMIS-PF: Patient-Reported Outcomes Measurement Information System-Physical Functioning.

i PROMIS-APS: Patient-Reported Outcomes Measurement Information System-Ability to Perform Social Roles and Activities.

j EQ-5D-3L: European Quality of Life 5 Dimensions 3 Level Version.

### Compliance

Of the 188 patients in the intervention group, 157 (83.5%) activated the app. In total, 155 patients (82.4%) answered the in-app screening questions, and 146 patients (77.7%) used the app at least once after first login. Therefore, 146 patients in the intervention group were included in the per-protocol analysis (Figure 2). The median number of days on which patients opened the app during the perioperative period was 25 days (IQR 7.5-39.0). When looking at the self-reported app use, 158 patients completed the questionnaire. A total of 91 patients (57.6%) indicated to have used the app regularly, 37 patients (23.4%) used the app occasionally, and 30 patients (19%) did not use the app.

### Physical Functioning

**Figure 3.**
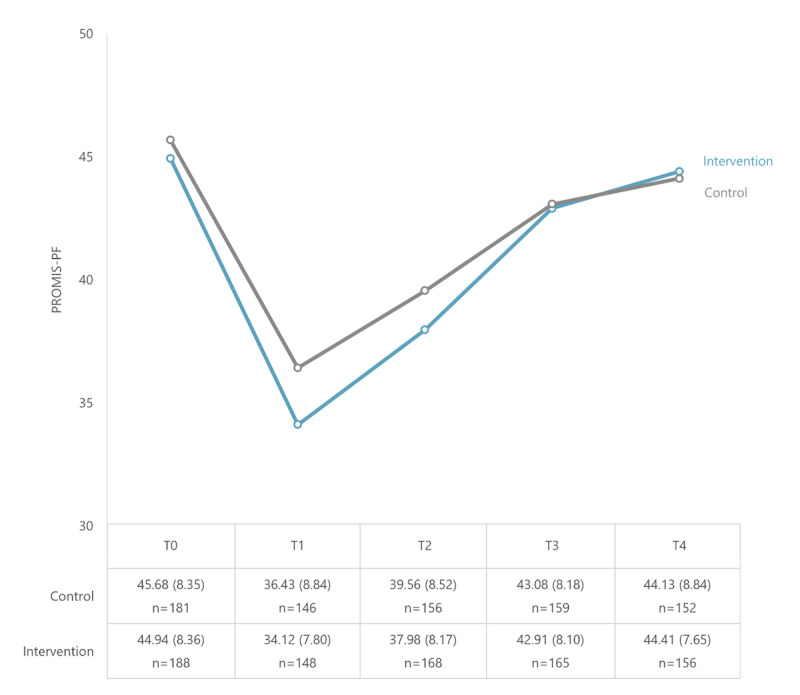
The course of PF (PROMIS-PF) across all time points (observed mean values and SDs). PF: physical functioning; PROMIS-PF: Patient-Reported Outcomes Measurement Information System-Physical Functioning; T0: before random assignment; T1: 1 week; T2: 3 weeks; T3: 6 weeks; T4: 12 weeks after hospital discharge.

The course of PF per group and the means and SDs per time point are shown in Figure 3. Results of the linear mixed effects models are shown in Table 3. The linear mixed effects models revealed that in both groups PF improved from 1 week after hospital discharge to 12 weeks after hospital discharge. The intervention group increased by 10.93 points over 12 weeks versus 7.96 points in the control group (time effect), resulting in a mean slope difference between groups (time by group interaction) of 2.97 over 12 weeks (95% CI 0.90-5.02; *P*=.005) in favor of the intervention group (Table 3 and Multimedia Appendix 2). The sensitivity analyses exploring the impact of missing data did not lead to different results. Imputation of missing data with the lowest score measured at that time point led to a mean slope difference of 2.60 (95% CI 0.53-4.65; *P*=.014) while imputation with the highest score led to a mean slope difference of 2.42 (95% CI 0.32-4.51; *P*=.023). In addition, sensitivity analysis for possible center effects showed that the time by group interaction was not different for each center (*P*=.749). The per-protocol analysis displayed similar results as the modified intention-to-treat analysis (mean slope difference=3.58, 95% CI 1.39-5.76; *P*=.001).

Per time point, the mean PF score at 1 week after hospital discharge (adjusted for baseline) was significantly lower in the intervention group than in the control group (mean slope difference=−1.72, 95% CI −3.38 to −0.07; *P*=.042). No significant differences at other time points were found (Multimedia Appendix 2).

**Table 3.** Mean slope differences between groups (time by group interaction) over 12-weeks for the primary and secondary longitudinal outcome measures.

	Intervention(n=188), mean	Control(n=181), mean	Mean slope difference between groups over 12 weeks (95% CI)	*P* value
Primary outcome				
CAT^a^ PROMIS-PF^b^ (version 1.2)^c^	10.92	7.95	2.97 (0.90 to 5.02)	.005^d^
Secondary outcome				
CAT PROMIS-APS^e^ (version 2.0)^c^	12.37	8.86	3.51 (−0.60 to 7.61)	.094
Self-reported functional recovery (0‐10)	3.29	2.63	0.66 (0.10 to 1.23)	.022^d^
EQ-5D-3L^f^ ^c^	0.16	0.14	0.02 (−0.03 to 0.08)	.391

a CAT: Computer Adaptive Test.

b PROMIS-PF: Patient-Reported Outcomes Measurement Information System-Physical Functioning

c Adjusted for baseline value of the outcome measure.

d Statistically significant *P*<.05.

e PROMIS-APS: Patient-Reported Outcomes Measurement Information System-Ability to Perform Social Roles and Activities

f EQ-5D-3L: European Quality of Life 5 Dimensions 3 Level Version

### Secondary Longitudinal Outcomes

No significant differences were found between groups on the postoperative recovery of social participation (3.51, 95%CI −0.60 to 7.61; *P*=.094) and health-related quality of life (0.02, 95% CI −0.03 to 0.08; *P*=.391). On the course of postoperative self-reported recovery (scale 0‐10), a significant difference was found favoring the intervention group (0.66, 95% CI 0.10 to 1.23, *P*=.022) (see Table 3).

The mean scores for social participation and self-reported recovery were significantly lower in the intervention group than in the control group at 1 and 3 weeks after hospital discharge, indicating a higher level of functioning in the control group. Social participation scores were 2.89 points (95%CI −4.93 to −0.86; *P*=.006) lower in the intervention group at 1 week and −3.12 points (95% CI −5.14 to −1.11; *P*=.003) lower at 3 weeks after hospital discharge. Self-reported recovery scores were −0.49 points (95% CI −0.98 to −0.01; *P*=.048) lower in the intervention group at 1 week and −0.58 points (95% CI −1.03 to −0.13; *P*=.012) lower at 3 weeks after hospital discharge. No significant differences at other time points were found for any of the secondary longitudinal outcomes (Multimedia Appendix 2).

### Postoperative Outcomes

No significant between-group differences were found for overall complications (OR 1.10, 95% CI 0.73-1.67; *P*=.658) and severe complications (OR 0.57, 95% CI 0.30-1.08, *P*=.085). In addition, no between-group differences were identified for length of stay (hazard ratio 0.95, 95% CI 0.78-1.17; *P*=.628), and hospital readmissions (OR 0.89, 95% CI 0.42-1.90; *P*=.764; Multimedia Appendix 2).

No significant differences were found for in-hospital physical and mental symptoms and mobilization (Multimedia Appendix 2). Preoperatively, a larger proportion of participants in the intervention group increased their muscle-strengthening activities compared to the control group (77% vs 35%; *P*<.001). Preoperative changes in other risk behaviors were similar for both the intervention and control groups (Multimedia Appendix 2).

### Patient Satisfaction

Participants in the intervention group gave a higher rating for satisfaction with overall perioperative care than participants in the control group (median 8.2, IQR 7.3-9.0 vs mean 7.8, IQR 6.6-8.7; *P*=.041). The median satisfaction score with the Be Prepared app was 8.1 (IQR 6.6-9.1). The Net Promotor Score for the Be Prepared app was 19, which can be considered good.

### Subgroup Analyses

**Figure 4.**
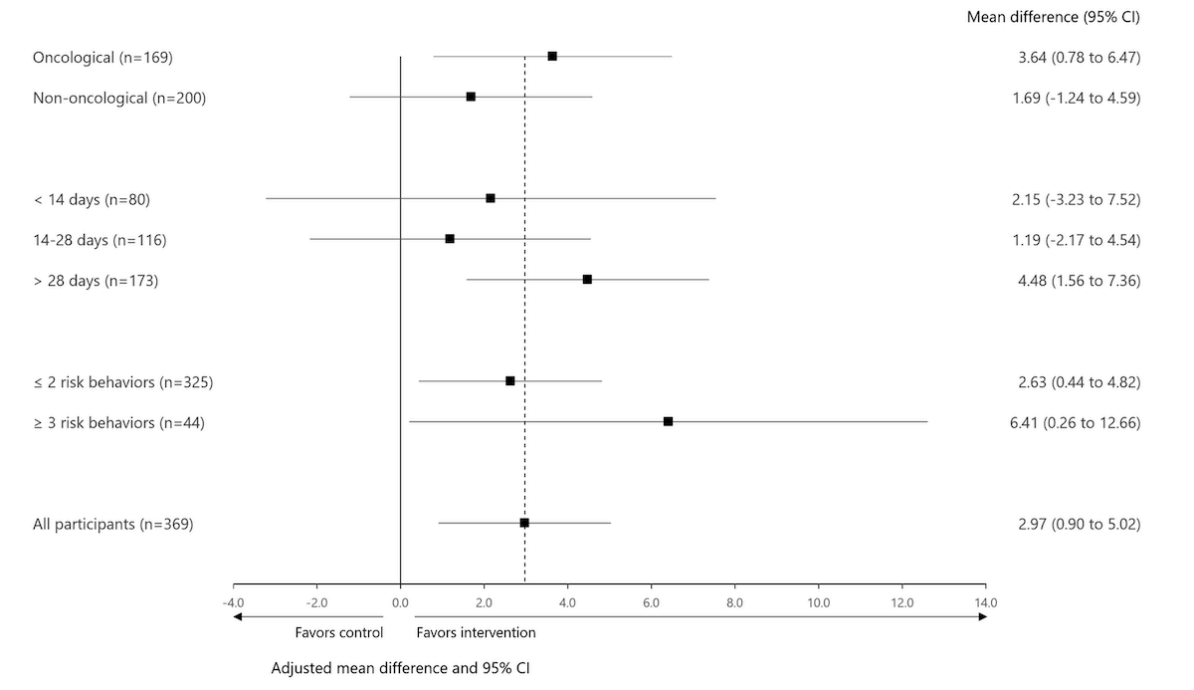
Forest plot of the subgroup analyses on the primary outcome of recovery of PF measured with Promis-PF. Bold line shows no effect point and dotted line shows overall treatment effect point. PF: physical functioning; PROMIS-PF: Patient-Reported Outcomes Measurement Information System-Physical Functioning.

Subgroup analyses on the primary outcome resulted in similar mean slope differences between groups (Figure 4). However, the subgroup analyses do show that treatment effects seem larger in some subgroups (ie, participants undergoing oncological surgery, participants with a preoperative preparation of more than 28 days, and participants with 3 or more risk behaviors).

## Discussion

### Principal Findings

In this multicenter RCT, the effectiveness of the Be Prepared mHealth intervention was evaluated in comparison with usual care in people undergoing major elective surgery. This study shows that the use of the Be Prepared app led to a small statistically significant improvement in the postoperative recovery of PF from 1 week to 12 weeks after hospital discharge, compared to patients receiving usual care. However, as this improvement is smaller than the estimated MID of 4.18, it can not be considered clinically meaningful. In addition, the intervention group showed a significantly greater decline in PF at 1 week after hospital discharge which seems to negate the effect on the postoperative recovery of PF. Most secondary outcome measures did not show significantly greater improvements in the intervention group compared to the control group, but patients in the intervention group scored significantly higher on self-reported recovery. Patient satisfaction with the overall perioperative care was significantly higher in the intervention than in the control group and participants in the intervention group expressed high satisfaction with the Be Prepared app.

Our hypothesis was that the Be Prepared intervention would improve the preoperative level of PF of patients and that they would therefore be able to better withstand the impact of major surgery, resulting in a smaller decrease of PF and a better and faster recovery after surgery. Our results, however, are in contrast with this hypothesis as the analysis of differences between groups at the individual postoperative time points (adjusted for baseline scores) showed that patients in the intervention group had a significantly lower level of PF at 1 week after hospital discharge than patients in the control group.

The limited effect found in this study could possibly be attributed to the characteristics of the Be Prepared intervention. First, the Be Prepared intervention was deployed as a stand-alone intervention without the involvement or guidance of health care professionals. Evidence suggests that supervised prehabilitation has a greater effect and higher adherence than unsupervised prehabilitation programs [5]. The Be Prepared intervention relies heavily on the initiative and discipline of the patient. In this study, only 58% of the patients indicated to have used the app regularly, and 19% did not use the app at all. With this evidence, it is questionable whether this approach, without the involvement of a health care professional, is sufficient to improve the postoperative course of recovery in every patient [29].

A second reason could be that the exercise component of the Be Prepared intervention does not provide sufficient stimuli to actually increase functional capacity and with that postoperative recovery. A recently published randomized clinical trial, the PREHAB trial, found beneficial effects on complications and postoperative recovery of a 4-week supervised multimodal prehabilitation program, consisting of high-intensity exercise, nutritional and psychological support, and smoking cessation, compared to usual care before colorectal cancer surgery [4]. While the Be Prepared intervention is also a multimodal intervention, the exercise component of the program focuses on increasing physical activity and muscle-strengthening activities according to the recommendations of the Dutch Health Council as opposed to high-intensity exercise training, as used in the aforementioned study. Given the current results, we can question whether the total stimulus in the Be Prepared app was sufficient to actually improve functional capacity, and with that improve the postoperative recovery in this patient group.

Third, within the Be Prepared app, no objective monitoring of progression, for example by activity trackers, was used for personalization and adjustment of treatment. Literature shows that adequate personalization at commencement of the program, and objective monitoring of progression to adjust treatment are essential [5,30]. Within the Be Prepared app, personalization was pursued based on the screening questions answered by the patient on commencement. Further adjustment of treatment based on progression was limited. For example, muscle strengthening exercises were scaled up or down based on patient-reported feedback in terms of perceived exertion on the proposed exercises in the app. The limited personalization and monitoring could have influenced adherence to the intervention and with that the effectiveness [29].

Besides the characteristics of the intervention, the selection of participants may also have played a role in the limited effect found in this study. The fact that mHealth might not be suitable for all patients [31,32] and that this suitability was not used as an inclusion criterion, may have resulted in the nonuse of the intervention and a smaller contrast between the groups than expected. Given this insight, a patient preference trial, instead of an RCT, might have been a better alternative [33,34].

Unfortunately, the COVID-19 pandemic impacted this study. The changed surgical planning resulted in longer waiting times or, on the contrary, short-term surgical planning, and surgeries were sometimes postponed several times. This might have impacted the effectiveness of the intervention as the app was not designed to adapt to changing surgery dates. As the information is presented in the app based on the surgery date, adjusting the surgery date would lead to the patient being presented with previously shown information.

The use of digital technologies is considered to have great potential and is an increasingly researched topic in the field of health care [10,11]. To our knowledge, this is the first study to perform a multicenter RCT evaluating the effect of a multimodal prehabilitation app on the postoperative course of PF in people undergoing major surgery. A recent systematic review on the use of digital technologies to support home-based prehabilitation prior to major surgery concluded that the use of technologies is feasible and has high acceptability [10]. However, none of the included studies evaluated postoperative recovery outcomes and the results of PF outcomes were inconclusive. A possible explanation for these inconclusive findings is the heterogeneity in the studied characteristics and outcomes, which hampers a clear comparison. For example, in our study, we used a smartphone app while other RCTs in this research area have mainly focused on telehealth or web-based interventions. Only one of the RCTs included in this review investigated a smartphone app as part of an eHealth intervention [35]. The main focus of the intervention was on managing recovery expectations and providing postoperative guidance. The study showed that the eHealth intervention was effective in decreasing the time taken to return to normal activities after intermediate-grade abdominal surgery. The magnitude of the effect on physical function over time, however, was comparable with the results found in our study.

An interesting finding, though one that is based on subgroup analyses, was the trend that patients with a more complex profile (patients with three or more risk behaviors and patients undergoing oncological surgery) seem to have a larger effect on the intervention. This argues for including especially high-risk patients for this type of intervention. In addition, patients with a preparation time longer than 28 days seemed to have a better effect of the intervention. Even though findings from previous research suggest that people’s engagement with technology wanes over time [36], the result from this subgroup analysis suggests that sufficient preparation time is important to reach an intervention effect. This also raises the question of whether the minimum intervention duration of 7 days in this study was adequate. The results of these subgroup analyses are all exploratory but can inform future research.

Despite the limited effect of the Be Prepared app on postoperative outcomes, patient satisfaction in the intervention group is high. Patients using the Be Prepared app are more satisfied with overall perioperative care and express high satisfaction with the app, which is an important finding as patient satisfaction is known to be a critical indicator of health care quality and the expected outcomes of care [14,37]. The usability and satisfaction with the app were explored using quantitative and qualitative data in the pilot RCT of the Be Prepared app [15]. In addition, the Be Prepared intervention can be considered a safe intervention for patients undergoing major elective surgery. Prior to using the Be Prepared mHealth intervention, patients were screened using the PAR-Q to determine whether they could safely participate in physical activity. Based on the PAR-Q results no individual adjustments to the intervention have been made. During the intervention, there was no supervision or monitoring by a health care professional and no intervention-related adverse events have taken place.

### Strengths and Limitations

This study has several important strengths, including a thorough process of designing, prototyping and evaluating the intervention, a large sample size, multicenter participation, and the use of both objective and self-reported outcomes.

Nevertheless, this study also has limitations.

First, almost a third of the eligible patients declined to participate in this study. While this is not unique and it is a commonly reported finding in (exercise) trials, it does raise questions about the generalizability of the results of this study to the target population [38]. As we could not collect any data on nonparticipants, except for reasons for nonparticipation, we are not able to compare their characteristics to the study participants.

Second, the engagement metrics available in this study were limited to basic usage logs, such as the date and time of app openings. Due to these limitations, we were unable to conduct a comprehensive analysis of app use which could have helped to understand the impact of app use on outcomes.

In addition, we did not reach our prespecified sample size and the dropout rate was higher than expected. Dropout could have introduced a selection bias in the results. Participants who only completed baseline questionnaires differed in their baseline score on the PROMIS-PF, CAT PROMIS-APS, and ASA classification, from participants who completed at least 1 follow-up. This may indicate that participants with a lower level of functioning were more likely to drop out.

In addition, given the increasing focus on the importance of preoperative preparation and prehabilitation in research, health care but also in mainstream media in the Netherlands, we cannot rule out the possibility that this has influenced usual care and diluted the contrast between the 2 groups in our trial, which could have led to an underestimation of the effect of the intervention.

### Conclusions

In this study, the Be Prepared app shows no added value regarding postoperative recovery and clinical outcomes. The Be Prepared app led to a statistically significant but not clinically relevant improvement in the postoperative recovery of PF up to 12 weeks after hospital discharge. In addition, we found a significantly greater decline in PF in the intervention group at 1 week after surgery. In light of these findings, implementation of the Be Prepared app as a stand-alone intervention does not appear to be beneficial for improving postoperative recovery. However, the Be Prepared app is a safe intervention, and patients using the Be Prepared app are more satisfied with overall perioperative care and express high satisfaction with the app. Given these benefits, mHealth can be considered a foundation for prehabilitation care pathways, but to be effective in improving postoperative recovery, preoperative mHealth apps should probably include objective monitoring and integration of mHealth with supervised prehabilitation according to patients’ needs and preferences. In addition, careful selection of patients with a preference for mHealth could increase adherence, and with that effectiveness.

## Supplementary material

10.2196/58703 Multimedia Appendix 1 Overview of the intervention content and features.

10.2196/58703 Multimedia Appendix 2 Supplementary tables.

10.2196/58703 Checklist 1 CONSORT-eHEALTH checklist (V1.6.1).

## Abbreviations

APS: Ability to Perform Social Roles and Activities
ASA: American Society of Anesthesiologists
CAT: Computer Adaptive Test
MID: minimal important difference
OR: odds ratio
PAR-Q: Physical Activity Readiness Questionnaire
PF: physical functioning
PROMIS: Patient-Reported Outcomes Measurement Information System
RCT: randomized controlled trial
