# Supplementary material for: The Effectiveness of the Be Prepared mHealth App on Recovery of Physical Functioning After Major Elective Surgery: Multicenter Randomized Controlled Trial
Source: JMIR Mhealth Uhealth. 2025 May 30;13:e58703. doi: 10.2196/58703 (PMC12143736; doi:10.2196/58703)
Supplement: Multimedia Appendix 1 [file mhealth-v13-e58703-s001.pdf]

Overview of the intervention content and features

Features and content	General preparation	Stress	Practical issues	Physical activity	Nutrition	Muscle strengthening	Alcohol cessation	Smoking cessation
Interactive timeline	✓	✓	✓	✓	✓	✓	✓	✓
Push notifications	✓	✓	✓	✓	✓	✓	✓	✓
Risk profile screening questions				< 30 minutes every day: Y/N	> 3kg unintentional weight loss last month: Y/N	< 2 days per week: Y/N	≥ 1 drink every day: Y/N	Currently smoking: Y/N
Information based on risk profile				✓	✓	✓	✓	✓
Preoperative content: from 24 days before surgery	✓	✓	✓	✓	✓	✓	✓	✓
Postoperative content: up to 30 days after surgery				✓	✓	✓	✓	✓
Text and photo content	Information	Information	Information<br>Tips	Information<br>Exercises<br>Tips<br>How and when to consult a physical therapist	Information<br>Link to external website<br>Tips<br>Protein rich recipes<br>How and when to consult a dietician	Information<br>Exercises<br>Tips<br>How and when to consult a physical therapist<br>Exercise library	Information<br>Tips<br>How and when to get extra help	Information<br>Did you know?<br>Tips<br>How and when to get extra help
Video content	Patient stories<br>HCP stories	Patient stories	Patient stories	Patient stories<br>HCP stories<br>Explainer video	HCP stories<br>Explainer video	HCP stories<br>Patient stories<br>Exercise videos	Patient stories<br>HCP stories	Patient stories<br>HCP stories
Interactive quiz questions		✓		✓	✓	✓	✓	✓
Behavior change techniques				Goal Setting<br>Provide information on consequences of behavior<br>Providing feedback on performance<br>Instructions on how to perform the behavior<br>Instructions about health consequences<br>Prompts/cues				
