# Supplementary material for: The Effectiveness of the Be Prepared mHealth App on Recovery of Physical Functioning After Major Elective Surgery: Multicenter Randomized Controlled Trial
Source: JMIR Mhealth Uhealth. 2025 May 30;13:e58703. doi: 10.2196/58703 (PMC12143736; doi:10.2196/58703)
Supplement: Multimedia Appendix 2 [file mhealth-v13-e58703-s002.docx]

**Table S1.** Model summary: linear mixed-effects model for PROMIS physical functioning.

| Predictor | Estimate | Standard error | *t* value |
| --- | --- | --- | --- |
| Intercept | 12.37 | 1.78 | 6.93 |
| Baseline CAT PROMIS-PF | 0.54 | 0.04 | 14.61 |
| Time per 12 weeks | 7.95 | 0.75 | 10.56 |
| Intervention group | –1.61 | 0.85 | –1.89 |
| Interaction time by intervention group | 2.97 | 1.05 | 2.82 |

*Abbreviations*

CAT PROMIS-PF = CAT PROMIS Physical Functioning.

**Table S2.** Estimated mean differences at each timepoint per outcome.

|  |  | Observed scores^a^ | | Estimated mean differences between groups^b^, | |
| --- | --- | --- | --- | --- | --- |
|  |  | Intervention group n=188 | Control group n=181 | MD (95% CI) | *P* value |
| Primary outcome |  |  |  |  |  |
| CAT PROMIS-PF (v1.2) | T0 | 44.94 (8.36) | 45.68 (8.35) | NA | NA |
|  | T1 | 34.12 (7.80) | 36.43 (8.84) | -1.72 (-3.38 to -0.07) | .042* |
|  | T2 | 37.98 (8.17) | 39.56 (8.52) | -1.02 (-2.59 to 0.55) | .205 |
|  | T3 | 42.91 (8.10) | 43.08 (8.18) | 0.70 (-0.74 to 2.15) | .342 |
|  | T4 | 44.41 (7.65) | 44.13 (8.84) | 1.24 (-0.25 to 2.74) | .106 |
| Secondary outcomes |  |  |  |  |  |
| CAT PROMIS-APS (v2.0) | T0 | 50.50 (11.08) | 49.91 (11.02) | NA | NA |
|  | T1 | 41.09 (7.30) | 43.86 (11.96) | -2.89 (-4.93 to -0.86) | .006* |
|  | T2 | 41.88 (6.84) | 44.59 (11.51) | -3.12 (-5.14 to -1.11) | .003* |
|  | T3 | 47.56 (11.39) | 48.98 (13.83) | -0.95 (-3.43 to 1.53) | .452 |
|  | T4 | 50.98 (14.46) | 51.28 (17.04) | 0.36 (-2.94 to 3.67) | .829 |
| Self-reported recovery (0-10) | T0 | NA | NA | NA | NA |
|  | T1 | 4.22 (2.07) | 4.77 (2.29) | -0.49 (-0.98 to -0.01) | .048* |
|  | T2 | 5.24 (2.11) | 5.75 (2.14) | -0.58 (-1.03 to -0.13) | .012* |
|  | T3 | 6.65 (2.07) | 6.71 (2.04) | 0.01 (-0.43 to 0.45) | .965 |
|  | T4 | 7.27 (2.01) | 7.30 (2.28) | 0.08 (-0.39 to 0.54) | .753 |
| EQ-5D-3L | T0 | .79 (.20) | .83 (.18) | NA | NA |
|  | T1 | .67 (.22) | .70 (.21) | -0.01 | .651 |
|  | T2 | .70 (.22) | .73 (.21) | -0.01 | .707 |
|  | T3 | .78 (.18) | .82 (.18) | -0.01 | .613 |
|  | T4 | .81 (.18) | .82 (.20) | 0.01 | .494 |

^a^Mean (SD).

^b^Adjusted for baseline value of the outcome measure

* Statistically significant *P*<0.05.

*Abbreviations*

CAT PROMIS-PF = CAT PROMIS Physical Functioning.

CAT PROMIS-APS = CAT PROMIS Ability to Perform Social roles and activities.

EQ-5D-3L = European Quality of Life 5 Dimensions 3 Level Version.

NA = Not applicable.

**Table S3.** Postoperative outcome measures: complications, length of stay and hospital readmissions.

| Outcome | Intervention n = 188 | | Control n = 181 | |  |  |
| --- | --- | --- | --- | --- | --- | --- |
|  | N | Data | N | Data | Estimate (95% CI) | *P* value |
| Complications, N (%) |  |  |  |  |  |  |
| Overall | 188 | 78 (41) | 181 | 71 (39) | OR 1.10 (0.73 to 1.67) | .658 |
| Severe^a^ | 188 | 17 (9) | 181 | 27 (15) | OR 0.57 (0.30 to 1.08) | .085 |
| Length of stay, median (IQR) | 188 | 5 (3 to 8) | 181 | 5 (3 to 7) | HR 0.95 (0.78 to 1.17) | .628 |
| Hospital readmission, N (%)^b^ | 188 | 14 (7) | 181 | 15 (8) | OR 0.89 (0.42 to 1.90) | .764 |

^a^Clavien Dindo classification ≥ grade III.

^b^Measured at 30 days.

**Table S4.** Postoperative outcome measures: in-hospital physical and mental symptoms, in-hospital mobilization.

| Outcome | Intervention n = 188 | | Control n = 181 | |  |
| --- | --- | --- | --- | --- | --- |
|  | N | Data | N | Data | *P* value |
| In-hospital physical symptoms (0-12), median (IQR) | 149 | 3.0 (1.0 to 5.0) | 147 | 3.0 (1.0 to 5.0) | .814 |
| In-hospital mental symptoms (0-16), median (IQR) | 149 | 3.0 (1.0 to 4.0) | 147 | 2.0 (0.0 to 5.0) | .266 |
| In-hospital mobilization (0-15), median (IQR) | 143 | 5.0 (4.0 to 8.0) | 142 | 5.0 (3.0 to 8.0) | .115 |

**Table S5.** Postoperative outcome measures: self-reported risk behavior change.

| Outcome | Intervention n = 188 | | Control n = 181 | |  |
| --- | --- | --- | --- | --- | --- |
|  | N | Data | N | Data | *P* value |
| Change in risk behavior, N (%) |  |  |  |  |  |
| Smoking | 14 | 10 (71) | 9 | 5 (56) | .103 |
| Alcohol consumption | 12 | 8 (40) | 6 | 5 (83) | .706 |
| Physical activity | 57 | 39 (68) | 64 | 43 (67) | .345 |
| Muscle strengthening  activities | 75 | 58 (77) | 80 | 28 (35) | <.001* |
| Protein rich food consumption | 11 | 9 (82) | 13 | 11 (85) | .609 |

* Statistically significant p < 0.05.
